# Effects of Electrostatic Field on Osteoblast Cells for Bone Regeneration Applications

**DOI:** 10.1155/2017/7124817

**Published:** 2017-11-13

**Authors:** Chen-Ying Su, Tzan Fang, Hsu-Wei Fang

**Affiliations:** ^1^Department of Chemical Engineering and Biotechnology, National Taipei University of Technology, 1, Sec. 3, Zhongxiao E. Rd., Taipei 10608, Taiwan; ^2^Institute of Biomedical Engineering and Nanomedicine, National Health Research Institutes, No. 35, Keyan Road, Zhunan Town, Miaoli County 35053, Taiwan

## Abstract

Many external stimulations have been shown to promote bone regeneration. The effects of an alternating current (AC) electrostatic field, one of external stimulations, generated from a device with high voltage and low current output on human osteoblastic cell line have been investigated in this study. We investigated how human osteoblasts responded to an AC electrostatic field, and the output parameters were set as 1 kV and 160 *μ*A. Our results showed that, under such condition, the AC electrostatic field had a downregulation effect on the production ability of alkaline phosphatase and type 1 collagen expression. However, the expression of osteocalcin gene was elevated on the end of EFID treatment suggesting that AC electrostatic field might be a potential stimulation for accelerating the differentiation of osteoblastic cells.

## 1. Introduction

Bone is rigid but can still be damaged by external pressure or joint diseases. As the damaged bones are repaired, renewal and reconstruction of bone tissues are in the progress. During bone formation, osteoblasts differentiate into osteocytes that are embedded in mineralized bone matrix [1]. Many external stimulations have been shown to affect bone growth and fracture repair, such as surface remodeling, chemical compounds, or electricity. Both culturing materials and layers of coating can be served as surface remodeling. When treating bone defects with collagen/hydroxyapatite scaffolds enriched with polycaprolactone, mesenchymal stem cells, and thrombocyte-rich solution, bone regeneration even occurred in vivo [2]. If the scaffolds were coated with calcium phosphate, there was a synergistic effect on bone regeneration in vivo suggesting that surface remodeling can promote bone growth [3]. Growth factors, biomolecules, or inorganic small molecules have also been used as chemical compounds to stimulate bone regeneration [4–6]. However, the effects of chemical compounds were not as obvious as surface remodeling.

Electricity is considered as a kind of physical stimulations. It has been shown that capacitively coupled electric field with direct current (DC) power source has been confirmed to have upregulating effects on calf peritoneum osteoblastic-like primary cells [7], MC3T3-E1 osteoblasts [8], and fetal bovine metacarpophalangeal joint chondrocytes [9] by detecting the expression of ALP, transforming growth factor *β*1 (TGF-*β*1), bone morphogenetic proteins (BMPs), aggrecan, proteoglycan, and type II collagen. The electromagnetic field had also been proven to be osteoinductive [10] and could upregulate the proliferation of human osteoblastic cells [11]. Recently, the pulsed electromagnetic field has been shown to stimulate human bone marrow stromal cells to proliferate and differentiate into osteoblastic cells [12].

However, studies using alternating current (AC) electrostimulations as an external stimulation are rarely reported. Utilizing a very low intensity AC power source has an inhibition effect on the proliferation of some cancer cells, implying that low intensity AC stimulation could be a new application in the treatment of tumor growth by reducing neoplasm cell division [13]. Thus it raised our interest in the bone fracture healing capability of electrostatic field induction device (EFID). In this study, we determined the effects of high voltage and low current on human osteoblastic cells by observing proliferating rate and the expression of osteocalcin and type I collagen for 28 days. Because different cell types demonstrate different proliferation rate, the proper initial cell densities should be taken into account. We then tested two different cell densities and found that the initial cell density did influence the differentiation of osteoblastic cells. Our study has provided the first evidence that AC electrostimulation might be a potential treatment for bone fracture healing.

## 2. Materials and Methods

### 2.1. Cell Cultures

The cells used in this study were human bone osteosarcoma cell line MG-63, purchased from the American Type Culture Collection (ATCC), and was initially derived from a 14-year-old Caucasian male. MG-63 cells were cultured in Minimum Essential Medium Eagle (MEM) containing 1.5 g/L sodium bicarbonate (NaHCO_3_) and seeded at two initial cell densities which were 1 × 10^4^ cells/well and 1 × 10^5^ cells/well.

### 2.2. Experiment Setup and EFID Application

The EFID was connected to one stainless steel plate, which was electrically insulated from the rest of the incubator. The installation of EFID has been previously described [14]. MG-63 cells were initially incubated in another incubator to a proper amount and were then harvested and seeded into 16 multiwell (24 wells) flat-bottom plates with two different initial densities. The plates loaded with MG-63 cells were next divided into two groups: experiment group with stimulation of electrostatic field generated by EFID and control group with no electrostatic stimulations.

**Figure 1:**
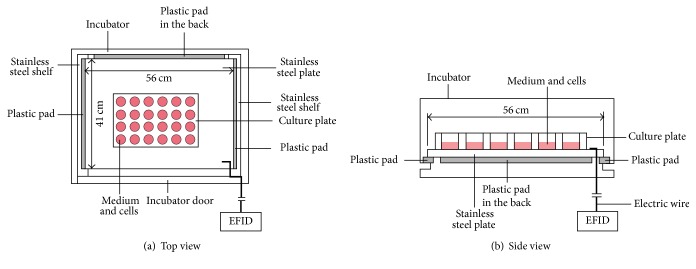
Schematic of electrostatic field induced device (EFID) installed in the cell culture incubator.

The multiwell plates in experiment group were placed on the insulated and wired stainless steel plate, and those in control group were placed on a stainless steel plate much lower than the one of experiment group. The stainless steel plate in control group was in contact with other parts of the incubator and was not electrified. The output parameters of the EFID used in this study were at its maximum power of 1.0 kV and 160 *μ*A. Cell cultures were continuously exposed to the electrostatic field generated by EFID. A schematic of the EFID setup in the cell incubator is shown in Figure 1.

### 2.3. Viability Assessment (MTT Assay)

3-(4,5-Dimethyl-2-thiazolyl)-2,5-diphenyl-2H-tetrazolium bromide (MTT) was purchased from SIGMA (product number M5655). The medium was discarded and 100 *μ*L of MTT solution was added to each well of 24-well plate. In the absence of light, the plate wrapped with aluminum foil was placed in the incubator for 3 hours. Then MTT solution was discarded and 200 *μ*L of dimethyl sulfoxide (DMSO) was added to each well and the plate was shaken gently for 15 minutes in the dark. When formazan precipitate was fully dissolved, the solution was transferred to 3 wells of an Enzyme-Linked ImmunoSorbent Assay (ELISA) plate to perform a triplicate test. The samples were then read by an ELISA reader with a wavelength of 570 nm. The results were showed in a form of optical density (OD) value.

### 2.4. Alkaline Phosphatase Activity (ALP Assay)

The culture medium was discarded, and cells were rinsed with PBS. 500 *μ*L of PBS containing 0.1 M glycine, 1 mM MgCl_2_, and 0.5% Triton X-100 (pH 10.5) was added to each well for 1 hour, and then 100 *μ*L of supernatant was transferred into a new tube wrapped with aluminum foil. 200 *μ*L of p-nitrophenyl phosphate solution (pNPP) (SIGMA, P7998) was added to each tube, and tubes were incubated at 37°C for 30 minutes, followed by incubated at 0°C for 10 minutes. The reaction was stopped with 50 *μ*L of 3 N NaOH solution, and solution was transferred to 3 wells of an ELISA plate to perform a triplicate test. The plate was read by using an ELISA reader with a wavelength of 405 nm.

### 2.5. Cell Morphology

The morphology of MG-63 cells was documented by photography. The Nikon optical microscope was equipped with a CCD digital camera. The camera was connected to a computer in which the software was installed. All the pictures taken by this equipment were added with a 100 *μ*m scale bar at the right lower corner to serve as a reference. Shuttle shape was the normal appearance of MG-63 cells. The cells should contain multiple turns and angles on the edge as well as a clear circular nucleus at the center of the cell. While differentiation proceeded, the cells would become thinner and longer.

### 2.6. Nitrite Index (Nitrite Production Ability)

A standard curve was prepared by using the 100 *μ*M sodium nitrite solution as stock, and 50 *μ*M, 25 *μ*M, 12.5 *μ*M, 6.25 *μ*M, 3.13 *μ*M, and 1.56 *μ*M solutions were obtained by diluting the stock solution with deionized water in a series of twofold dilutions. 50 *μ*L of each concentration of sodium nitrite solution was added to 3 wells of an ELISA plate to perform a triplicate test. 50 *μ*L of culture medium was transferred to one well of ELISA plate in triplicate followed by addition of 50 *μ*L of sulfanilamide solution (C_6_H_8_N_2_O_2_S, 1% sulfanilamide in 5% phosphoric acid). The samples were then incubated at room temperature for 10 minutes in the dark, and 50 *μ*L of NED solution (C_12_H_14_N_2_, 0.1% N-1-napthylethylenediamine dihydrochloride in H_2_O) was added at room temperature in the dark for 10 minutes. A purple or magenta color would be formed immediately, and the plate was read by using an ELISA reader at 546 nm wavelength within 30 minutes.

### 2.7. Gene Expression Analysis

Total ribonucleic acid (RNA) was isolated by using the Trizol purification system according to the manufacturer's instructions (Invitrogen). Reverse transcription (RT) of total RNA to single-stranded complementary deoxyribonucleic acid (cDNA) was completed by using the high-capacity cDNA reverse transcription kits (Applied Biosystems) as described.

Gene expression was analyzed by real-time polymerase chain reaction (PCR) using an ABI SteponePlus sequence detection machine (Applied Biosystems). Glyceraldehyde-3-phosphate dehydrogenase (GAPDH: endogenous control), type I collagen (COL1A1), and osteocalcin (OCN) were evaluated in this study, and the design of primer sequences was shown in Table 1. A cycle threshold value (Ct) was obtained from each sample and triplicate sample values were averaged. The 2^−ΔΔCt^ method was then used to calculate the relative expression of each target gene.

### 2.8. Statistical Analysis

The statistical analysis was accomplished with the aid of computer statistical software MINITAB® Release 14. All the statistical analysis was performed with one-way ANOVA method under a 95% confidence level and the calculated *P* value represented the difference level. When *P* value was smaller than 0.05, there was a significant difference between two targets and was marked as one asterisk in the figures. When *P* value was smaller than 0.005, two asterisks were marked in the figures.

## 3. Results and Discussion

### 3.1. Low Cell Density with and without EFID

**Figure 2:**
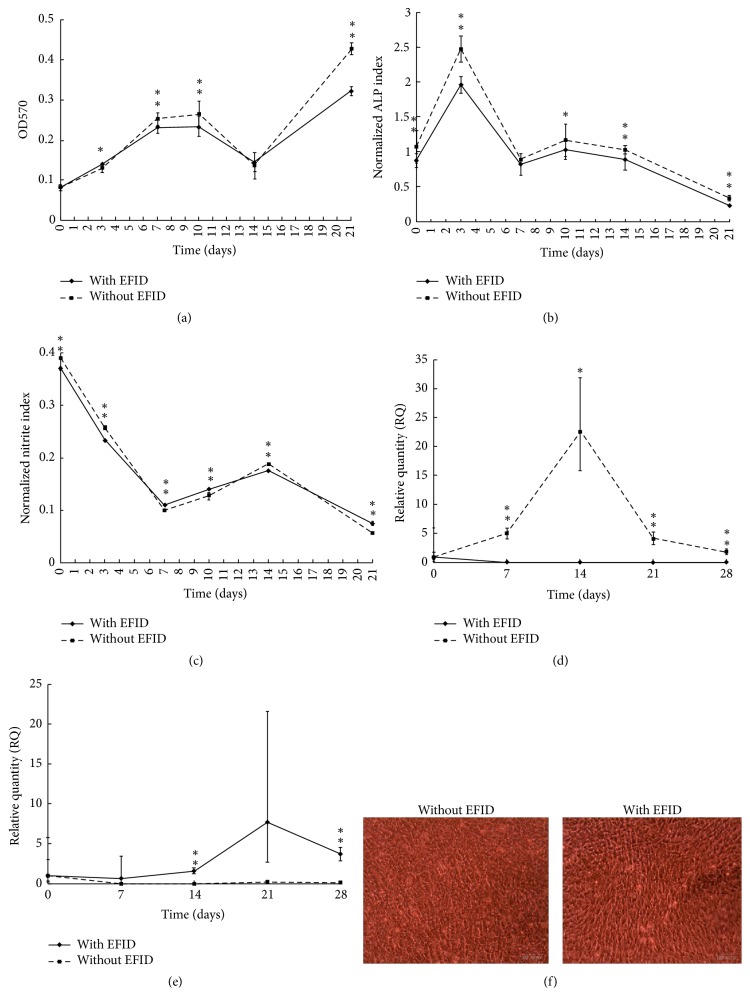
(a) Overall cell viability with and without EFID stimulation at 1 × 10^4^ cells/well. (b) ALP production ability per cell with and without EFID stimulation at 1 × 10^4^ cells/well. (c) Nitrite production ability per cell per day with and without EFID stimulation at 1 × 10^4^ cells/well. (d) COL1A1 gene activity per cell with and without EFID stimulation at 1 × 10^4^ cells/well. (e) OCN gene activity per cell with and without EFID stimulation at 1 × 10^4^ cells/well. (f) Cell morphology at day 28 without (left) and with (right) EFID stimulation at 1 × 10^4^ cells/well. ^*∗*^*P* < 0.05 and ^*∗∗*^*P* < 0.005.

Figure 2 illustrated the results of analysis when low cell density was treated with or without EFID. The cell viability was significantly greater without the introduction of EFID (Figure 2(a)), and the ALP production was significantly reduced regardless of the treatment of EFID (Figure 2(b)). The nitrite production index was significantly lowered with EFID treatment although it was higher with EFID treatment at days 7 and 14 compared to no-EFID treatment (Figure 2(c)). The EFID showed significant reduction effect on COL1A1 gene activity, suggesting the type I collagen content in extracellular matrix (ECM) may be reduced (Figure 2(d)). However, the OCN gene activity was raised at day 14 and day 28 with EFID treatment (Figure 2(e)). The results of cell morphology were similar in EFID-treated and no-EFID groups after culturing for 28 days (Figure 2(f)).

There are three major stages for osteoblastogenesis, including proliferation, matrix maturation, and mineralization [15]. The most used markers for the process of osteoblastogenesis are ALP, bone sialoprotein, and COL1A1 as early markers of osteoblast differentiation while parathyroid hormone (PTH)/PTH-related protein receptor and OCN appear later during mineralization [16]. At an initial cell density of 1 × 10^4^ cells/well, the external electrostimulation from EFID clearly reduced the viability, ALP production ability, and COL1A1 gene activity. The proliferation of osteoblasts was likely to be slowed down with EFID, except at day 3 (Figure 2(a)). The stimulation duration was thought to be a factor that regulated the proliferation and maturation of osteoblasts. A research report stated that when using DC capacitively coupled electric field, max expression of inducible BMPs always occurred after 24 h of stimulation [17]. It may imply that the upregulation effect of external electrostimulation may be time dependent. In this study, it was possible that the AC electrostimulation had positive effects in cell viability within a short period of time such as 1-2 days but regulated proliferation negatively when the stimulation time was more than 2 days.

The COL1A1 activity was significantly higher without EFID application, suggesting richer type I collagen content in ECM. Thus higher cell viability and ALP activity were observed in the control group. The EFID reduced the proliferation of MG-63 cells but did not inhibit mineralization because the expression of OCN was increased (Figure 2(e)). It has been postulated that the development of osteoblasts depended on free Ca^2+^ concentration within 1.2–1.8 mM, and small deviation inhibited the activity [18]. The Ca^2+^ concentration in culture media may be a factor that regulated the expression level of OCN genes, but it was difficult to determine the actual Ca^2+^ concentration in the media since any tiny disturbance of pipetting could result in turbulence vortex. Therefore, whether Ca^2+^ concentration has an effect on OCN expression with EFID treatment requires further investigation.

### 3.2. High Cell Density with and without EFID

**Figure 3:**
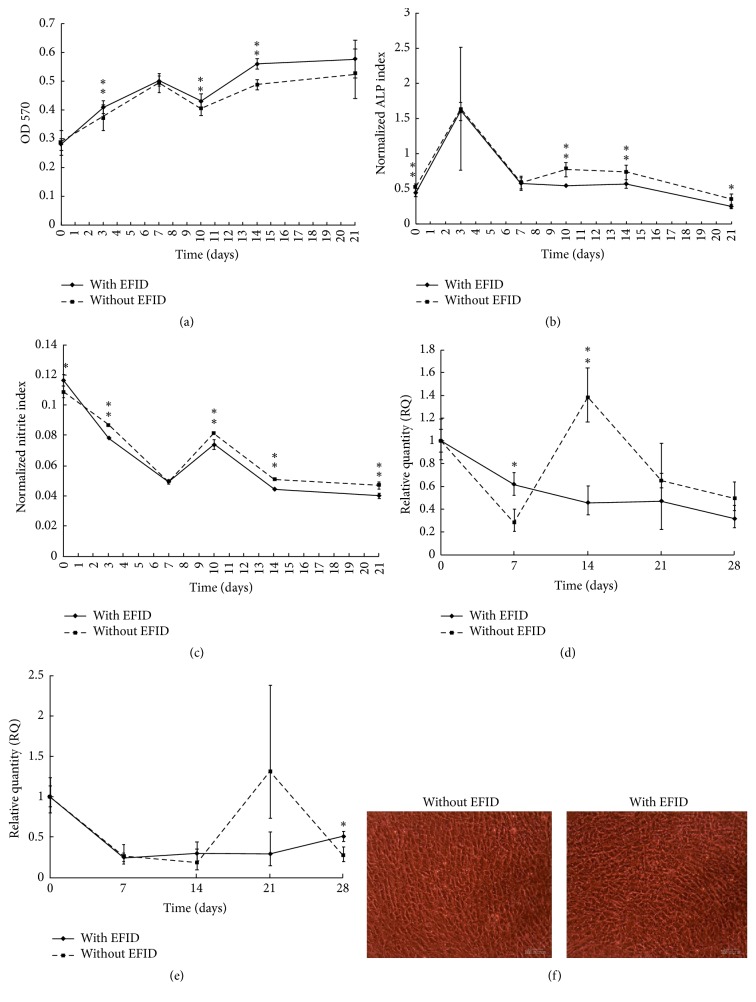
(a) Overall cell viability with and without EFID stimulation at 1 × 10^5^ cells/well. (b) ALP production ability per cell with and without EFID stimulation at 1 × 10^5^ cells/well. (c) Nitrite production ability per cell per day with and without EFID stimulation at 1 × 10^5^ cells/well. (d) COL1A1 gene activity per cell with and without EFID stimulation at 1 × 10^5^ cells/well. (e) OCN gene activity per cell with and without EFID stimulation at 1 × 10^5^ cells/well. (f) Cell morphology at day 28 without (left) and with (right) EFID stimulation at 1 × 10^5^ cells/well. ^*∗*^*P* < 0.05 and ^*∗∗*^*P* < 0.005.

Figure 3 illustrated the results of analysis when high cell density was treated with or without EFID. The cell viability was significantly greater with the presence of EFID (Figure 3(a)), but the ALP production ability was reduced in a great extent with EFID compared to control, especially after one week of incubation (Figure 3(b)). The nitrite production index was lowered with EFID treatment after day 3 (Figure 3(c)), as well as the COL1A1 expression after day 7 (Figure 3(d)). The expression of OCN was reduced with EFID treatment, except at days 14 and 28 (Figure 3(e)). The results of cell morphology were similar in EFID-treated and no-EFID groups after culturing for 28 days (Figure 3(f)).

Cells at higher initial seeding density in general tended to proliferate without much differentiation when EFID was introduced. Indeed, the activity of ALP and expression of COL1A1 and OCN were lower with EFID treatment. Only the viability of cells was higher with treatment of EFID, suggesting that cells tend to stay undifferentiated at high density. Therefore, we next compared all the analysis when the initial cell density was 1 × 10^4^ cells/well and 1 × 10^5^ cells/well.

### 3.3. Comparison of Different Initial Cell Density with EFID on Osteoblastic Cells

**Figure 4:**
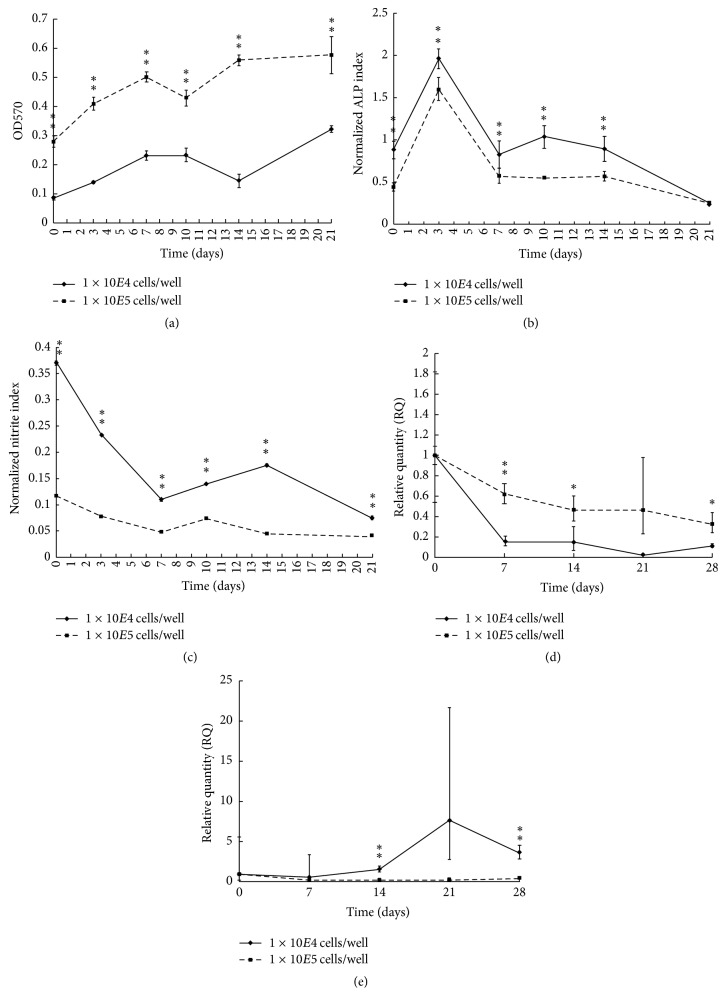
(a) Overall cell viability with EFID stimulation at 1 × 10^4^ cells/well and 1 × 10^5^ cells/well initial cell densities. (b) ALP production ability per cell with EFID stimulation at 1 × 10^4^ cells/well and 1 × 10^5^ cells/well initial cell densities. (c) Nitrite production ability per cell per day with EFID stimulation at 1 × 10^4^ cells/well and 1 × 10^5^ cells/well initial cell densities. (d) COL1A1 gene activity per cell with EFID stimulation at 1 × 10^4^ cells/well and 1 × 10^5^ cells/well initial cell densities. (e) OCN gene activity per cell with EFID stimulation at 1 × 10^4^ cells/well and 1 × 10^5^ cells/well initial cell densities. ^*∗*^*P* < 0.05 and ^*∗∗*^*P* < 0.005.

Figure 4 illustrated the results of analysis with EFID at 1 × 10^4^ cells/well and 1 × 10^5^ cells/well initial cell densities. The cell viability was significantly higher in an initial cell density of 1 × 10^5^ cells/well throughout the entire experiment period (Figure 4(a)). The ALP production ability per cell was significantly reduced with higher initial cell density (Figure 4(b)), as well as the nitrite production index (Figure 4(c)) indicating the proliferation condition of osteoblasts was better with a higher initial cell density. The COL1A1 gene activities of both groups were reduced with the application of EFID, but the situation was worse in 1 × 10^4^ cells/well group (Figure 4(d)). The application of EFID immediately inhibited COL1A1 gene activity with a lower initial cell density, resulting in a sharp drop from day 0 to day 7 while COL1A1 gene activity in 1 × 10^5^ cells/well group was gradually decreased. The OCN gene expression in 1 × 10^4^ cells/well group was increased but remained low in 1 × 10^5^ cells/well group (Figure 4(e)).

**Figure 5:**
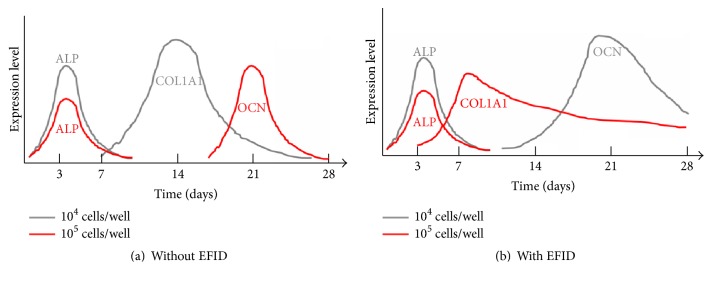
(a) In the absence of EFID, a lower initial cell density expresses more COA1A1 while a higher initial cell density tends to differentiate and expresses higher OCN. (b) In the presence of EFID, a lower initial cell density stimulates to differentiate (high OCN expression) while a higher initial cell density remains to establish extracellular matrix (high COL1A1 expression).

The summary was shown in Figure 5, and a higher initial cell density with EFID treatment resulted in higher cell viability and COL1A1 expression. In contrast, a lower initial cell density with EFID treatment led to higher ALP expression, the nitrite production, and OCN expression. Our results suggested that a higher initial cell density under the influence of electrostatic field facilitated cells to remain proliferated and establish the ECM. In contrast, a lower initial cell density with EFID stimulated cells to differentiation for matrix maturation and mineralization. Normally, higher cell density leads to high differentiation efficiency [19–21]. However, the outcome was opposite with EFID stimulation. It is possible that EFID provides an electric orientation for osteoblasts to migrate, which is a critical movement coupled with mineralization. While cell density is high, collagen-containing ECM is well established; thus there is no sufficient space for osteoblasts to migrate resulting in the reduction of mineralization and differentiation. The two-dimensional* in vitro* testing environment is not the best condition for mimicking the in vivo biological process; in particular bone formation requires three-dimensional mineralized matrix to guide the growth and differentiation of osteoblasts [22]. In order to understand whether the mineralization and migration of osteoblasts are affected by increased cell density with the stimulation of EFID, culturing osteoblasts in a three-dimensional scaffold will be needed.

Regardless of initial cell density, we observed higher OCN expression in EFID-treated group than non-EFID-treated group (Figures 2(e) and 3(e)). Some evidences have indicated that the secretion of bone morphogenic proteins (BMPs) in osteoblasts was upregulated by capacitively coupled electric field; in particular, the expression of BMPs 2, 4, 5, 6, 7, and 9 was increased [17]. BMPs 2, 4, 5, 6, and 7 have osteogenic capacity, and many studies have shown that those BMPs can induce the differentiation of osteoblasts [23–28]. It is possible that the EFID we used in this study may promote the expression of BMPs, directing osteoblasts to undergo differentiation. Therefore, the underlying molecular mechanisms by which EFID stimulates differentiation of osteoblasts into matrix maturation will require further investigation.

The electromagnetic fields have been shown to affect cell cycle on cells. After applying low frequency electromagnetic field onto human epidermal stem cells, the percentage of cells in the DNA synthesis stage was increased and in the G1 phase was decreased indicating the electromagnetic field can promote proliferation [29]. Another research has demonstrated that after bone marrow mesenchymal stem cells were exposed to pulsed electromagnetic field, the proportion of cells in the newly divided cells was increased while cells in the mitosis phase were decreased [30]. Our results showed that EFID could promote proliferation at higher initial cell density. However, continuous stimulation of EFID seemed to direct cells to establish ECM or differentiate into matrix maturation and mineralization suggesting that cells would leave cell cycle under our experimental condition. Previous studies have only focused on a short-term effect of electromagnetic fields (up to 7 days), but our results provided long-term effects (28 days) of electrostatic field on human osteoblastic cells.

## 4. Conclusions

The effect of the electrostatic field induction device (EFID) on human osteoblastic cells was investigated, and two different initial cell densities were taken into account. The results showed that the expression of osteocalcin was elevated with EFID treatment suggesting EFID can stimulate differentiation of osteoblasts. With the stimulation of EFID, a lower initial cell density demonstrated better differentiation capacity while higher initial cell density showed better establishment of extracellular matrix. This outcome may contribute to providing a potential treatment for bone regeneration.

## Figures and Tables

**Table 1:** Primer information for real-time PCR.

Gene	Accession number	Sequence	Melting temperature (°C)
GAPDH	NM_002046	Forward: 5′-GAACATCATCCCTGCCTCTACTG-3′ Reverse: 5′-TCCGACGCCTGCTTCACC-3′	58.2 58.8

COL1A1	NM_000088	Forward: 5′-CAGGGCGACAGAGGCATAAAG-3′ Reverse: 5′-CCAGCCATTGATACAGGTAGC-3′	58.3 58.3

OCN	NM_000711	Forward: 5′-GCAGAGTCCAGCAAAGGTG-3′ Reverse: 5′-CCAGCCATTGATACAGGTAGC-3′	55.5 55.7
